# How the stomach and the brain work together at rest

**DOI:** 10.7554/eLife.37009

**Published:** 2018-05-04

**Authors:** Giuseppina Porciello, Alessandro Monti, Salvatore Maria Aglioti

**Affiliations:** 1 Department of PsychologySapienza University of RomeRomeItaly; 2 IRCCS Santa Lucia FoundationRomeItaly

**Keywords:** resting-state networks, phase synchrony, body maps, gastric rhythm, gastric network, Human

## Abstract

Low-frequency electrical waves in the stomach seem to be synchronised with the activity of a newly discovered resting-state network in the human brain.

**Related research article** Rebollo I, Devauchelle AD, Béranger B, Tallon-Baudry C. 2018. Stomach-brain synchrony reveals a novel, delayed-connectivity resting-state network in humans. *eLife* **7**:e33321. doi: 10.7554/eLife.33321

The brain is always active – even when it is at rest, it receives a continuous stream of information from other areas of the body. From gut feelings to heartbeats, this information is constantly monitored to maintain a state of physiological equilibrium known as homeostasis. Signals from the body, including the stomach, also influence a variety of mental processes and complex human behaviours (Critchley and Harrison, 2013; Herbert and Pollatos, 2012; Porciello et al., 2018). Although the anatomy of the homeostatic neural pathway is relatively well known (Craig, 2003), its physiology is less well understood.

During periods of wakeful rest, our brain generates its own spontaneous and synchronised activity within different groups of brain regions (known as resting-state networks). On the other hand, specialised cells in the stomach produce a slow, continuous pattern of electrical impulses that set the pace of stomach contractions during digestion. But the stomach also generates these signals when it is empty, which suggests that they may have another purpose. Now, in eLife, Ignacio Rebollo of the PSL Research University and co-workers – Anne-Dominique Devauchelle, Benoît Béranger, and Catherine Tallon-Baudry – report how they have combined two techniques, functional magnetic resonance imaging and electrogastrography, to shed new light on the interactions between the brain and the stomach (Rebollo et al., 2018).

Rebollo et al. placed electrodes on the abdomen of volunteers as they lay inside a brain scanner and analysed the coupling between the signals from the stomach and the brain using a method called phase-locking value analysis. The researchers discovered a new resting-state network – the gastric network – which fired in synchrony with the rhythm of the stomach (Figure 1A). Furthermore, the various brain regions within this network showed a delayed functional connectivity between each other.

**Figure 1.**
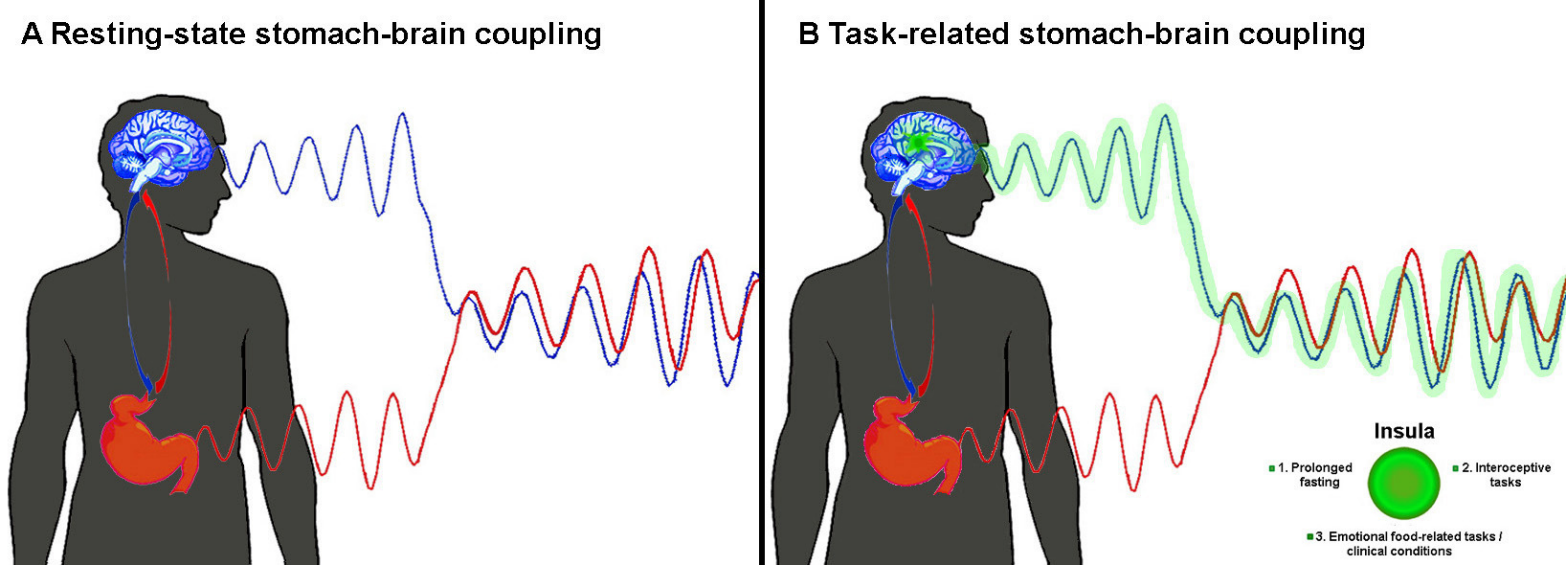
Coupling between the activity of the brain and the stomach. (**A**) When the body is at rest, Rebollo et al. found that the blood flow in the brain (as measured by functional magnetic resonance imaging; blue wave) and the electrical activity in the stomach (as measured by electrogastrography; red wave) are in delayed sync with each other (Rebollo et al., 2018). (**B**) Rebollo et al. discovered that the insula (green) was only marginally coupled with the stomach. We think that the activity of this region (green wave) will be more synchronous with that of the stomach when certain conditions are met (see main text).

Although the role of the gastric network remains unknown, Rebollo et al. reasoned that some of the brain regions within the network map information about vision, touch and movement in bodily coordinates. Thus, they advanced the tantalising hypothesis that this network coordinates different ‘body-centred maps’ in the brain. According to this, a region in the brain known as the insula should be strongly involved in the gastric network: this region receives direct input from the internal organs (e.g. stomach, intestine), which is integrated to create a coherent representation of the whole body (Critchley and Harrison, 2013; Craig, 2009). However, Rebollo et al. found that the insula was only marginally synchronised to the rhythm of the stomach.

Thus, we suggest that the gastric network may rather act as a homeostatic regulator of food intake. Indeed, it neatly overlaps with areas processing information from the face, mouth and hands, and with three brain regions activated by tongue- or hand-related actions (Amiez and Petrides, 2014). We speculate that the insula would play a bigger role in the network if at least one of the following applied: the fasting happened over a longer period; the participants had to complete tasks that made them focus on their own gut feelings (interoceptive tasks); the participants attached a higher emotional value to food, either as a source of reward or disgust, as happens in people with eating disorders such as anorexia or bulimia nervosa (Figure 1B).

Due to the limitations of the phase-locking method (Lachaux et al., 1999), it remains unclear if the rhythmic interaction between the stomach and the brain is one-directional or two-directional. Clarifying this issue and measuring the stomach-brain coupling during conditions in which the body is far from homeostasis, and during interoceptive or emotional tasks, may help to shed light on the true functional role of the gastric network. We believe that the findings of Rebollo et al. not only open new avenues to improving our understanding of the resting-state activity, but also fire up an exciting debate on how signals from the enteric nervous system in the gut could shape the brain.
